# A Synopsis of Proceedings of the Texas State Medical Association

**Published:** 1888-05

**Authors:** F. E. Daniel

**Affiliations:** Secretary


### Society Notes.

### A SYNOPSIS OF PROCEEDINGS

*Texas State Medical Association.*

The Twentieth Annual Convention Texas State Medical Association was held in Galveston April 24th, 25th, 26th and 27th ult. There were about 200 physicians in attendance. The meetings took place in Harmony Hall, and the Sections met in various parts of the city. The exhibits, of which there were quite a number, were displayed in the hall of the Tremont Hotel.

FIRST DAY, TUESDAY, APRIL 24.—MORNING SESSION.

The meeting was called to order by Chairman J. F. Y. Paine, of the Arrangement Committee. Prayer by Rev. J. D. Scott, Pastor St. Johns church.

Dr. Paine introduced Hon. R. L. Fulton, Mayor of Galveston, who welcomed the Association in behalf of the city.

Major F. Charles Hume was then introduced and delivered an eloquent address on behalf of the citizens. He was followed by Dr. H. A. West, Chairman of the Reception Committee, in a flowery and ardent welcome in the name of the profession of the city.

Dr. Sam R. Burroughs, the President, was then presented, and in a beautiful speech responded to the many and cordial words of welcome in the name of the Association.

The President delivered the annual address, conveying certain recommendations and suggestions; referred to a committee, consisting of R. H. Harrison, Sr., Geo. Cupples, J. C. Jones, E. M. Rabb and W. P. Burts.

Dr. J. F. Y. Paine read his report as Chairman of the Committee of Arrangements.

On call of Judicial Council the following members were present: M. H. Oliver, W. A. Archer, R. C. Nettles, W. L. York, P. C. Coleman, M. D. Knox, H. H. Thorpe. Absent: P. Jordan, J. R. Johnson, D. M. Ray, R. Rutherford. Dr. T. J. Tyner asked to be excused from the Council on the ground that he had served three years. The Chair appointed *pro tem.* to the vacancies—J. T.

Field, S. D. Thruston, F. H. Tucker, A. M. Curtis and M. A. Taylor. Adjourned.

AFTERNOON SESSION, 3 P. M.

Reading of minutes of last meeting dispensed with on motion of Dr. Talley.

The report of the Committee on Constitution and By-Laws having been made the special order of the day, was called for, and read by Chairman Talley. Report received, on motion, and committee discharged with thanks of the Association. Report referred to committee appointed to consider the recommendations made by the President. Adjourned.

[The afternoons were devoted to Section work.]

SECOND DAY, WEDNESDAY, APRIL 25, 10 A. M.

Minutes of previous meeting read and approved.

The address of the Chairman of Section on Surgery was then read, Dr. A. W. Fly, Chairman, and referred to the Publishing Committee.

The Judicial Council made report on applications for new membership. Thirty-two new members were admitted as follows:

Castleton, E. S. E., ........ Houston.
Smith, Bat ........ Wharton.
Raysor, P. M., ........ Chappell Hill.
Hodge, R. C., ........ Galveston.
Duke, Jno., ........ Alvarado.
Miller, J. W., ........ Massey, Hill Co.
Savage, W. S., ........ Rogers Prairie.
Magruder, F. B., ........ Seeley.
Autrey, A. M., ........ Houston.
Martin, W. A., ........ Cottondale.
Gibson, W. F., ........ Livingston.
Tucker, J. P., ........ Oeerton.
Watson, J. S., ........ Burnet.
Thrash, D. E., ........ Galveston.
Baker, Z. W., ........ Temple.
Fisher, W. C., ........ Galveston.
Parsons, E. B., ........ Moscow.
Johnson, R. A., ........ Flatonia.
Johnson, J. C., ........ Cold Spring.
Monroe, D., ........ Cameron.
Sherman, T. M., ........ Coltharp.
Howze, Jno. T., ........ Huntsville.

Kaiser, F. W.,........................................Flatonia.
Matthews, W. C.,......................................Kauffman.
Abney, O. L.,.........................................Victoria.
Montgomery, A. S.,....................................Tyler.
Pope, Irvin,..........................................Tyler.
Lee, Geo. H.,.........................................Galveston.
Dillard, Jno. L.,.....................................Richmond.
Stone, Sam. A.,.......................................Richmond.
Spring, John V.,......................................San Antonio.
Wolff, Arthur S.,.....................................Brownsville.

HONORARY MEMBERS.

T. Moore Madden,......................................Dublin, Ireland.
Jas. L. Cabell,.......................................Charlotteville, Va.

They also reported as represented by delegate, the following associations in affiliation: Dallas county, Hill county, Terrell, and Waco Medical Societies.

Val Verde Medical Society was admitted to affiliation on application.

Dr, R. W. Knox, chairman of Section on Electro-Therapeutics, read his address, which, on motion, was received with thanks and referred to Publishing Committee.

Report of Section on Obstetrics, etc., being next on the programme, was called for, but the chairman, Dr. Dial, being reported sick, and the Secretary, Dr. Musick, absent, it was passed.

Dr. Ramsdell, chairman of Section on Practice, was absent, also, and the chairman of the Section on Public Hygiene and State Medicine, Dr. R. M. Swearingen, made a brief verbal report, saying he had not had opportunity or inclination to make an address, inasmuch as so little interest seemed to be taken in the subject by members; he had not received a single paper.

The chairman of the Committee of Arrangements read an invitation from the Mayor, requesting the members to visit the Artesian wells at their convenience, and stating that an excursion by rail to the Jetties in the bay, had been arranged for that afternoon. Invitations accepted with thanks.

[The Jetties were visited by about 150 members, and many ladies of delegates' families.]

The address in the Section of Dermatology was called for. Dr. Dudley, the chairman, was absent.

On motion, the President appointed Dr. Lowry Chairman, and Dr. Williams, Secretary, and the papers in that Section were placed in their hands and subsequently read in Section.

Dr. T. D. Wooten, chairman of the committee appointed some years ago to secure for the use of the Association a room in the capitol at Austin, made a verbal report, stating that there were several rooms vacant, and that one or more could be had on application, and recommended that the Association make such application. Report received, and a committee appointed, on motion of Dr. Christian, to memorialize the Legislature on the subject. The Chair appointed Drs. F. R. Martin, A. L. Cocke, H. H. Thorpe, R. S. Gregg and G. W. Christian.

[The original committee, all being residents of Austin, declined to serve.]

Adjourned.

THIRD DAY, THURSDAY, APRIL 26.

9:30 a. m.—President Burroughs in the chair. On motion, reading of the minutes dispensed with.

The chairman of the Section on Practice, etc., Dr. C. M. Ramsdell, read his report or address, which was referred to the Publishing Committee.

The report was briefly discussed, and in the discussion Drs. Lowry, Talley, C. F. Paine and others, gave their formulæ for the use of Carbolic Acid, Cocaine, etc., and on motion of Dr. Talley, the Publishing Committee were requested to publish these formulæ in the transactions, as a part of the proceedings.

[The Secretary requested to be furnished with copies for publication, but up to date none have been received, except from Dr. Lowry.]

Dr. Cupples offered a resolution that a telegram be sent to Dr. Clopton, expressing sympathy in his misfortune, and regret at his enforced absence from the meeting. Amended by Dr. Oliver to include a similar telegram to Dr. S. H. Stout, who had the misfortune to suffer dislocation of the shoulder, which prevented his attendance. Carried, and telegrams were sent by the President.

Dr. Cupples, chairman of the Committee on Surgical Cases, etc., presented his bill for postage on report, on circulars, etc., for $31.50, which was approved and paid.

The Bell county Medical Association, which was not formally represented at last meeting, having declined to pay the pro rata tax, applied to be re-admitted to affiliation. Approved by Judicial Council.

Dr. G. W. Christian, chairman of the Section on Gynæcology, read his address. On motion, it was referred to Publishing Committee. [No discussion.]

The Secretary read an invitation from the Mayor and Council of San Antonio to hold the next meeting of the Association in that city ; also, similar invitations from the Mayors and Councils of Waco and of Paris. Referred to Nominating Committee.

Dr. H. A. West, chairman of the Section on Medical Jurisprudence, Chemistry, etc., made his report. Referred to Publishing Committee, and on motion of Dr. Lowry, a vote of thanks was tendered Dr. West.

Dr. West's paper dealt with the subject of insanity before the courts, and was listened to with deep interest. Dr. Ramsdell said he was glad Dr. West had presented the paper; it was a subject too much neglected by physicians; they seemed afraid to facc it. It was a subject upon which improved legislation is needed, and the legislators should be enlightened on insanity from a modern standpoint. He had often wondered that the laws allow an insane person to go at large after being so adjudged. It was an easy matter for any criminal to "go insane," and thus escape justice. If the laws were more strict and the gallows was the alternative, there would be less feigned insanity.

Dr. Sears never could see why a crazy criminal should not be hung, if adjudged guilty by the jury. He should submit to it the more cheerfully.

Dr. E. L. Ward said the paper of Dr. West was one of the most interesting and absorbing that he had ever heard. If he had not known Dr. West, he would have taken him for a preacher, when he began his paper; a little later he thought he was a poet; and as he got well into the intricacies of the vexed question and made all so clear, he was convinced that West was a lawyer. The laws are too lax, and should be renovated; but any improvement in this regard must emanate from the medical profession, who are alone competent to frame laws adapted to the case under discussion, and we have learned physiologists and psychologists who should be consulted by the legislators as to the requrements on this head. Dr. Ward felt incompetent to thoroughly discuss the important subject as presented by Dr. West's able paper, and called for Dr. D. R. Wallace.

[Superintendent of the Insane Asylum at Terrell, Dr. Wallace, was absent.]

Dr. Talley said the subject under discussion was one of the most important a physician has to deal with. The paper, he said, was well written and well read. The most important point in connection with the subject is, the physicians are themselves responsible for the abuses of the criminal-insane law. They make it a point to keep out of court; don't want to go there. Wallace has been telegraphed for to Waco now, to give expert testimony in similar cases. He is one who is not afraid. Lawyers are shrewd, and often, judges of human nature. They will feel around by asking questions in social intercourse, and find a doctor to testify the way they want him to testify; seeking *not the truth*, but the triumph of their side. This is why, Dr. Talley said, that he advocated that expert testimony should be on the part of the court, and not in behalf of any individual.

Dr. O. L. Williams cut off further discussion by saying members were complaining that the general session was encroaching on the time allotted to Section work.

Dr. Paine, chairman of the Arrangement Committee, here read a number of letters from distinguished medical men all over the world, in answer to invitations to be present; among them one from Dr. T. Moore Madden, of Dublin, enclosing a contribution (Ovarian Hernia) to Section of Gynæcology; one from J. Matthews Duncan, London; from Thomas Bryant, and others. All referred to the Secretary, to be preserved in the archives.

Dr. B. A. Pope, chairman of the Section on Ophthalmology, read his address. On motion, it was referred to the Publishing Committee.

The Nominating Committee was announced, and retired.

[The Section on Gynæcology, by invitation, held its session in the hall, after the general session; Dr. G. W. Christian, chairman, presiding.

Dr. George Lee read Dr. Madden's paper, "Ovarian Hernia," which, on motion of Dr. Ghent, was referred to the Publishing Committee, and the Secretary instructed to return the thanks of the Association to the author.

[The paper was discussed by Dr. F. T. Paine and others, but we are obliged to omit his remarks, as well as nearly all discussion. We can give here only a synopsis of the proceedings.—ED.]

Several other papers were read in this Section.

[Most of the papers read in the Sections are in the hands of the Secretary; some are not, some have been withdrawn, and he has received *no report of any discussions* from the Secretaries of some Sections.]

The papers contributed to the several sections, and which are in the hands of the Publishing Committee, are as follows:

### SECTION ON PRACTICE OF MEDICINE, ETC.

Report of Chairman, C. M. Ramsdell, A. M., M. D., Lampasas:

1. A case of Sclerosis of the Liver: By Irwin Pope, M. D., Tyler.

2. A case of Malarial Purpura Hemorrhagica: By J. C. J. King, M. D., Waco.

3. Staining of Bacilli Tuberculosis: By T. E. Nott, M. D., Chattanooga, Tenn. [Read by caption, and referred to Publishing Committee.]

[A paper on The Modern Treatment of Neuralgia: By Professor Landon Carter Gray, of the New York Polyclinic, has been received by the Secretary, it having reached Galveston after the meeting adjourned.]

### SECTION ON OBSTETRICS AND DISEASES OF WOMEN.

[No report by Chairman received by publishing Committee, and no papers of any description.]

### SECTION ON SURGERY AND ANATOMY.

Report of Chairman, A. W. Fly, M. D., Galveston:

1. Case of Embolism of Femoral Artery: By O. Eastland, M. D., Wichita Falls.

2. Mammary Hypertrophy: By George H. Nelson, M. D., Terrell.

3. Compound Fracture of the Skull: By E. T. Walker, M. D., Trinity.

4. Several cases of Extensive Burns: By C. M. Ramsdell, M. D., Lampasas.

5. Case of Traumatic Lockjaw: By C. H. Wilkinson, M. D., Galveston.

6. Report of a case of Fractured Patella: By W. G. Jameson, M. D., Rusk.

7. Extensive Fracture of the Skull; Trephined: By B. F. Brittain, M. D., Jacksonville.

8. The Silicate of Soda Splint, or fixed Bandage: By J. W. Carhart, M. D., Lampasas.

SECTION ON MEDICAL JURISPRUDENCE, ETC.

Report by the Chairman, H. A. West, M. D., Galveston:
[No papers reported to the General Secretary.]

SECTION ON GYNECOLOGY.

Report by Chairman, G. W. Christian, M. D., Burnett:

1. Treatment of Diseases of the Womb: By John Inabnit, M. D., Terrell.

2. Displacement of the Ovaries: By T. Moore Madden, M. D., Dublin, I.

SECTION ON OPHTHALMOLOGY AND OTOLOGY.

Report by Chairman, B. A. Pope, M. D., Dallas:
[In hands of author for revision. He has also the papers read in his Section.]

SECTION ON DERMATOLOGY.

[No report by Chairman has been received.]

1. A Plea for Early Surgical Interference in Epithelioma of the Skin, etc.: By Daniel Parker, M. D. Calvert.

2. Eczema and its Lesions: By J. D. Bass, M. D., Pittsburg.

3. Some Intractable Skin Diseases: C. M. Ramsdell, A. M., M. D., Lampasas.

SECTION ON ELECTRO-THERAPEUTICS.

Report by the Chairman, R. W. Knox, M. D., Houston.

1. Clinical Notes on Galvanism: By C. M. Ramsdell, A. M., M. D., Lampasas.

2. Treatment of Trichiasis by Electrolysis: By Daniel Parker, M. D., Calvert.

3. Effects of Electric Current in Nausea and Vomiting of Pregnancy, etc.: By O. L. Williams, M. D., Chapel Hill.

---

GENERAL SESSION—3 P. M., THURSDAY, APRIL 26.

---

Vice-President Knox presiding.

The Nominating Committee made their report as follows:

REPORT OF NOMINATING COMMITTEE OFFICERS ELECT, 1888.

President, J. F. Y. Paine, Galveston.

First Vice-President, H. K. Leake, Dallas.

Second Vice-President, A. V. Doak, Taylor.

Third Vice-President, O. Eastland, Wichita Falls.

*Judicial Council.*—New members, I. E. Clark, Schulenburg; C. M. Ramsdell, Lampasas; T. J. Bell, Tyler; R. H. Harrison, Sr., Columbus.

*Section on Practice.*—Chairman, R. B. White, Ennis; Secretary, R. A. McCall, Ennis.

*Section on Obstetrics.*—Chairman, W. H. Wilkes, Waco.

*Section on Surgery and Anatomy.*—Chairman, J. C. Jones, Gonzales.

*Section on Gynecology.*—Chairman, B. E. Hadra, Austin.

*Section on State Medicine and Public Hygiene.*—Chairman, R. M. Swearingen, Austin.

*Section on Medical Jurisprudence, Psychology and Chemistry.*—Chairman, D. R. Wallace, Terrell.

*Section on Ophthalmology and Otology.*—Chairman, R. H. Chilton, Dallas.

*Section on Electro-Therapeutics.*—Chairman, W. M. Powell, Albany.

*Section on Dermatology.*—Chairmam, H. L. Taylor, Waco.

*Publishing Committee.*—F. E. Daniel, Secretary, Austin, Ex-officio Chairman; R. M. Swearingen, Austin; J. W. McLaughlin, Austin.

*Committee of Arrangements.*—Chairman, F. Herff, San Antonio.

*Delegates to American Medical Association, at Newport, Rhode Island, June, 1889.*—J. F. Y. Paine, B. F. Kingsley, C. H. Wilkinson, J. C. Jones, E. J. Beall, J. W. McLaughlin, H. C. Ghent, R. T. Knox, A. W. Fly, J. H. Sears, S. D. Thruston, G. W. Christian, W. W. Reeves, D. C. Hewson, N. A. Olive, A. M. Douglas, B. Sanders, George Cupples, T. J. Tyner, F. Allen, O. L. Williams, R. M. Swearingen, C. F. Paine, T. D. Wooten, L. B. Creath, A. C. Walker, E. P. Becton, E. T. Walker, A. B. Gardner (of Belleville), J. W. Garnett, M. S. Crow.

*Delegates to Alabama State Medical Association.*—J. D. Osborn, H. C. Ghent.

*Representative to the British Medical Association, in September, 1888.*—T. J. Tyner, Austin.

Dr. T. Moore Madden, 55 Merrian Square, Dublin, and Professor J. L. Cabell, Charlottsville, Va., were made honorary members.

Dr. William Caston's resignation was received, the Doctor being about to remove from the State.

Dr. Cupples, chairman of the Committee on Surgical Cases, made his report, which, on motion, was read and referred to Publishing Committee. (Recalled by the author.) Adjourned.

FOURTH DAY, FRIDAY, APRIL 27.—MORNING SESSION.

President Burroughs in the chair. Reading of the minutes dispensed with.

The Secretary, Dr. F. E. Daniel, read his report and also that of the Publishing Committee. Received and referred to committee of three, Drs. Tyner, Hadra and Q. C. Smith.

Treasurer report read and received also, and the usual appropriation was voted him.

Bills for secretary salary and for the special appropriation for the Publishing Committee were approved and paid.

The Committee on Prize Essays made their report. Three papers had been presented. After a careful examination, the committee were of the opinion that the one on "The Opthalmoscope in General Practice," and bearing the motto "Excelsior" was the best and recommended it for the prize. Report received and committee discharged. The paper was, after much discussion as to the interpretation of the law, referred to a special committee of Ophthalmologists for examination and decision. The chair appointed Drs. G. P. Hall, R. C. Hodges, of Galveston, and B. A. Pope, of Dallas. The committee will report at next meeting.

The newly elected officers were installed, and made appropriate speeches, Dr. Burroughs surrendering the gavel in a neat little address.

The Committee on Necrology had no report.

Dr. Cupples, on behalf of the delegation to the International Congress made a verbal report.

Delegates to other associations made no reports.

Dr. Harrison, chairman of the committee to whom was referred the President's recommendations, and the report of the Committee on Constitution and By-Laws made a report, but as the meeting was nearing a close, and the subject one of much importance, it was, on motion, recommitted and postponed till next meeting for final action. Adjourned.

AFTERNOON SESSION.

Dr. M. A. Taylor, chairman of the committee appointed last meeting to draft a circular letter to the people of Texas, setting forth the necessity of a law to regulate the practice of medicine, made a report, which was received, and on motion the circular letter gotten up and made a part of the report, was ordered published in the Galveston News. [It will appear in the transactions.]

Dr. Ghent offered a resolution earnestly requesting the legislature to appropriate $150,000 for the establishment of the Medical Department of the State University, and addressed the meeting at length on the subject. At the conclusion Dr. Robertson offered the following substitute, which was carried :

"*Resolved*, That it is the opinion of the Texas State Medical Association that the interest of the people of Texas requires the putting into operation of the Medical Branch of the State University without delay."

After electing Dr. Madden, of Dublin, Ireland, and Dr. Cabell, Charlottesville, Va., honorary members, and adopted the usual series of resolutions of thanks, ect., the Association adjourned to meet in San Antonio on the 4th Tuesday in April, 1889.

The social features of the meeting were most enjoyable ; a reception, a ball and a grand banquet at night, and an excursion by rail, and a sail on the bay, constituted a most enjoyable programme.

F. E. Daniel, Secretary.

